# Tuberculosis of the Thyroid Gland Presented as a Rapid Enlargement of a Preexisting Goiter

**DOI:** 10.1155/2018/4369531

**Published:** 2018-11-12

**Authors:** Ibtissem Oueslati, Imen Sakka, Olfa Ismail, Ines Akrout, Adel Marghli, Melika Chihaoui

**Affiliations:** ^1^Department of Endocrinology, La Rabta hospital, Faculty of Medicine, University of Tunis El Manar, Tunis, Tunisia; ^2^Department of Pathology, Abderrahman Mami Hospital, Ariana, Tunisia; ^3^Department of Pneumology, Abderrahman Mami Hospital, Ariana, Faculty of Medicine, University of Tunis El Manar, Tunis, Tunisia; ^4^Department of Thoracic Surgery, Abderrahman Mami Hospital, Faculty of Medicine, University of Tunis El Manar, Tunis, Tunisia

## Abstract

Thyroid involvement with tuberculosis is an uncommon condition even in endemic countries. As its clinical presentation is not specific, diagnosis is often difficult and requires histopathological confirmation. Herein we report an observation of secondary tuberculosis of the thyroid gland in a woman with a type 2 diabetes mellitus and a primary hypothyroidism. She presented with a rapid enlargement of a preexisting goiter without compressive symptoms. The imaging exams showed a voluminous plunging multinodular thyroid gland and multiple bilateral lung nodules. Malignancy was suspected and the patient underwent a total thyroidectomy and a lung biopsy. Histopathological examination revealed multiple tuberculous foci involving both the thyroid gland and the lungs.

## 1. Introduction

Both primary and secondary tuberculosis of the thyroid gland are rare. This is explained by the gland resistant mechanisms [1]. In fact, it has been shown that the bactericidal property of the colloid material, the high vascularity of the gland, and the presence of iodine are involved in the thyroid resistance to tuberculosis [2].

The first case of primary thyroid infection was reported in 1893 by Bruns [3]. It presented as a rapidly enlarging goiter with cervical lymphadenopathy without any evidence of pulmonary tuberculosis. Then, only few cases have been reported, especially in countries with high tuberculosis prevalence. The exact prevalence of the infection is lacking, varying from 0.1 to 1.15% [4, 5]. However, it seems that its incidence is increasing as a result of the routine practice of fine-needle aspiration cytology.

Herein, we report a new case of tuberculosis of the thyroid gland in a 48-year-old woman with type 2 diabetes, a primary hypothyroidism, and a preexisting goiter. It was probably a reactivation of a latent pulmonary infection.

## 2. Case Report

A 48-year-old woman presented with a rapid enlargement of a preexisting goiter without compressive symptoms. Her past medical history included type 2 diabetes mellitus, hypertension, goiter, and primary hypothyroidism for fifteen years. There was no past or present history of smoking and her family history was unremarkable.

She was complaining of productive cough for two weeks. However, she did not have any history of fever, night sweats, or anorexia.

Clinical examination showed a normal body temperature, a body mass index of 35.88 kg/m^2^, a blood pressure of 120/80 mmHg, a regular pulse of 89 beats/min, and a normal respiration rate of 20 breaths/min. The lung breath sounds were normal without any rales being heard. Cervical examination revealed a plunging multinodular goiter without any lymphadenopathy. Other systemic and regional examinations did not show any abnormalities.

The blood routine tests showed a fasting blood glucose of 7.19 mmol/l, a plasma creatinine level of 49 *μ*mol/l, a C-reactive protein level of 5 mg/l (reference range < 5 mg/l), an erythrocyte sedimentation rate of 57 mm/first hour, a red blood cells count of 4.38 *∗* 10^6^/mm^3^, a total hemoglobin concentration of 12.8 g/dl, a white blood cells count of 6800/mm^3^, a neutrophil count of 3640/mm^3^, and a lymphocyte count of 2220/mm^3^. Liver function tests were normal.

The thyroid function tests disclosed normal serum thyroid stimulating hormone (TSH) level at 0.5 *μ*IU/ml (reference range: 0.35-4.94) and normal free thyroxin (FT4) level at 9.14 pmol/L (reference range: 8.5-25) on daily 100 *μ*g of levothyroxine.

**Figure 1:**
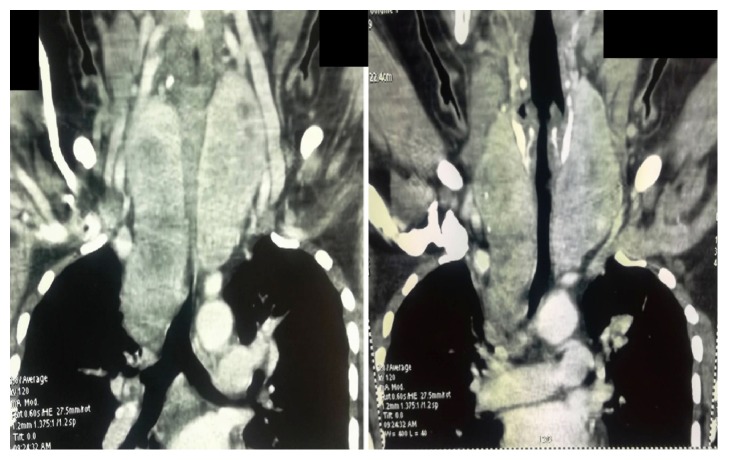
Cervical computed tomography scan revealed an enlarged plunging multinodular thyroid gland.

Thyroid ultrasound showed a heterogeneous multinodular goiter. Her chest X-ray showed a mediastinal enlargement and a suspicious lesion located at the upper lobe of the right lung. Cervical and chest computed tomography scan revealed an enlarged plunging multinodular thyroid gland (right lobe: 113 × 39 × 41 mm, left lobe: 90 × 53 × 44 mm) with an extension of the right lobe into Barety's space (Figure 1) and multiple bilateral lung nodules.

Sputum smear microscopy was negative.

Thyroid cancer was suspected. Fine needle aspiration (FNA) cytology was not available for technical reasons. Surgical treatment was indicated and the patient underwent a total thyroidectomy. Multiple lung biopsies were also performed using a left anterior minithoracotomy through the fifth intercostal space.

**Figure 2:**
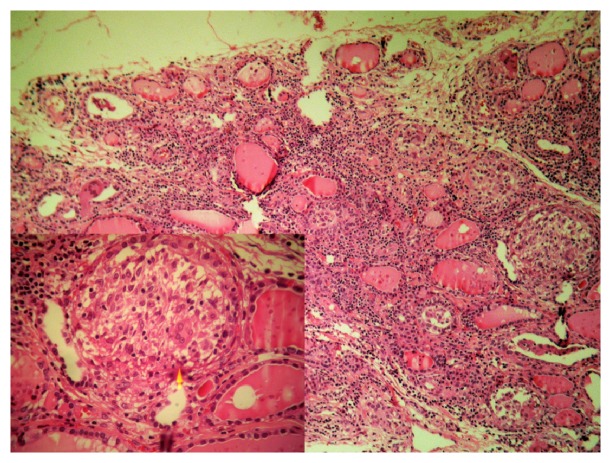
Granulomas in the thyroid (HEx100): the arrow shows epithelioid and langhans giant cells (HEx400).

**Figure 3:**
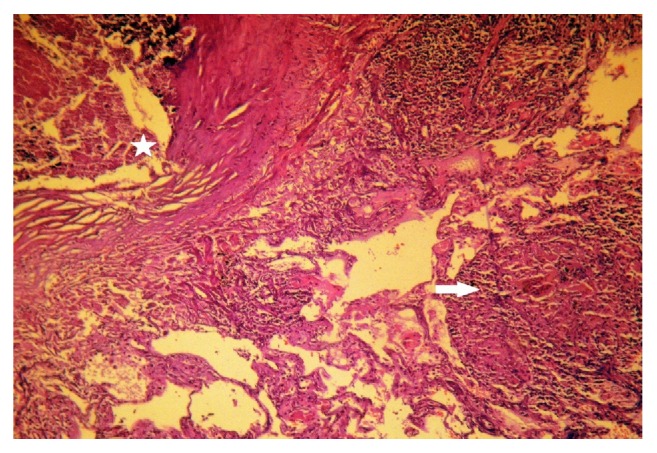
Pulmonary tuberculosis with caseous necrosis (asterisk) and confluent tuberculosis granulomas (arrow) (HEx200).

Histopathological examination showed a benign multinodular hyperplasia with epithelioid cell granulomas and giant cells (Figure 2). These morphological signs were compatible with multiple tuberculous foci of the thyroid gland. The histopathological examination of the lung biopsies showed foci of granulomatous inflammation along with caseous necrosis (Figure 3).

The diagnosis of tuberculosis involving both the lung and the thyroid gland was established and the patient was treated with antituberculosis drugs for 6 months.

## 3. Discussion

To our knowledge, this is the fourth case of tuberculosis of the thyroid gland reported in our country [6–8]. All cases were female patients aged 49, 56, and 68 years. In the literature, the mean age at onset was around the third to the fourth decades with a slight female predominance [2].

The thyroid primary involvement of the disease is a very rare condition [9]. In fact, tuberculosis of the thyroid gland generally results from miliary spread as a part of generalized dissemination or via direct extension from cervical lymph nodes or from the larynx [10, 11]. The majority of reported cases were accompanied by other extra-thyroid loci of the disease. The coexistence of pulmonary tuberculosis as in our patient was described in only few cases [12].

Some risk factors such as age, diabetes mellitus, malnutrition, and acquired immunodeficiency syndrome were associated with the occurrence of tuberculosis of the thyroid gland [13, 14]. Our patient was at risk for developing tuberculosis as she had a chronic disease which may affect the immune system. In fact, diabetic patients have an overall threefold increased risk of developing active tuberculosis [15].

Tuberculous infection of the thyroid gland may present with several clinical manifestations. Our patient presented with a recently rapid enlargement of a preexisting goiter without compressive symptoms. In most published cases, the clinical presentation was subacute. Acute onset was rather described in case of an abscess or a thyroiditis [16, 17]. Five different clinical presentations have been described: goiter with caseation, cold abscess formation, acute abscess, miliary tuberculosis, and chronic fibrosing tuberculosis [12, 18].

The constitutional symptoms of tuberculosis such as fever, weight loss, night sweats, and anorexia may be present [1]. In our case, we suspected a recent reactivation of latent pulmonary tuberculosis because of the absence of active disease manifestations such as fever, night sweats, and weight loss. Our patient complained of productive cough for two weeks that could be the first tuberculosis reactivation symptom.

Although thyroid function remained generally normal, some cases with hyperthyroidism or hypothyroidism were reported. In fact, thyrotoxicosis may occur in early stages as a result of thyroid cells destruction and the release of thyroid hormones into the circulation. The occurrence of hypothyroidism is explained by extensive glandular destruction by caseous necrosis.

Our patient had a primary hypothyroidism treated with levothyroxine for 15 years. Its most likely etiology is Hashimoto's thyroiditis.

Routine investigations remain normal except for a raised erythrocyte sedimentation rate as in our case [19].

The imaging techniques are not very helpful for the diagnosis of tuberculosis of the thyroid gland. Neck ultrasound may reveal round heterogeneously hypoechoic unique or multiple lesions or anechoic lesion, irregular borders of nodules, and regional adenopathy. On computed tomography scan, a round centrally hypodense mass with peripheral enhancement may be seen [20].

In our case, tuberculosis of the thyroid gland was weakly suspected before surgery. Because of the rapid enlargement of the preexisting goiter and the presence of thyroid and lung nodules, a malignant tumor was strongly suspected. In such cases, fine-needle aspiration cytology should be considered as it has been shown to be useful to detect tuberculosis [5, 9] and to rule out the diagnosis of other inflammatory diseases and malignancy. It can avoid unnecessary surgery.

Main differential diagnosis of thyroid tuberculosis is De Quervain's thyroiditis and sarcoidosis. It can be difficult if caseous necrosis and extrathyroid tuberculosis are absent. In our case, the tuberculous involvement of the thyroid was established when the concomitant pulmonary tuberculosis was confirmed.

The treatment of tuberculosis of the thyroid gland is based on antituberculosis drugs [10, 21]. Surgery is only indicated in case of a large thyroid abscess warranting a surgical drainage or the removal of a part of the gland.

After total thyroidectomy, an antituberculosis treatment for at least 6 months is administrated in patients with additional tuberculosis foci [9]. However, if other foci are not detected, a close follow-up with no additional antituberculosis treatment is recommended [9]. If tuberculosis is found in patients who have undergone subtotal or near-total thyroidectomy or thyroid lobectomy, an antituberculosis treatment of at least 6 months is administered, regardless of the presence of any additional foci [9].

## 4. Conclusion

Although thyroid tuberculosis is a rare condition, it should be considered as a differential diagnosis of thyroid masses especially in an endemic country. In this case, the use of fine-needle aspiration cytology could help to avoid unnecessary surgical intervention.
